# A study on nickel application methods for optimizing soybean growth

**DOI:** 10.1038/s41598-024-58149-w

**Published:** 2024-05-08

**Authors:** Bruna Wurr Rodak, Douglas Siqueira Freitas, Monica Lanzoni Rossi, Francisco Scaglia Linhares, Edemar Moro, Cid Naudi Silva Campos, André Rodrigues Reis, Luiz Roberto Guimarães Guilherme, José Lavres

**Affiliations:** 1 Department of Agronomy, São Paulo Western University, Presidente Prudente, São Paulo 19067-175 Brazil; 2 Center for Nuclear Energy in Agriculture, University of São Paulo, Piracicaba, São Paulo 13416-000 Brazil; 3 Department of Agricultural and Natural Sciences, State University of Minas Gerais, Ituiutaba, Minas Gerais 38302-192 Brazil; 4 Chapadão of Sul Campus, Federal University of Mato Grosso of Sul, Chapadão do Sul, Mato Grosso do Sul 79560-000 Brazil; 5 School of Science and Engineering, São Paulo State University, Tupã, São Paulo 17602-496 Brazil; 6 Department of Soil Science, Federal University of Lavras, Lavras, Minas Gerais 37200-000 Brazil

**Keywords:** Plant development, Plant sciences, Plant symbiosis, Rhizobial symbiosis

## Abstract

Fertilization with nickel (Ni) can positively affect plant development due to the role of this micronutrient in nitrogen (N) metabolism, namely, through urease and NiFe-hydrogenase. Although the application of Ni is an emerging practice in modern agriculture, its effectiveness strongly depends on the chosen application method, making further research in this area essential. The individual and combined effects of different Ni application methods—seed treatment, leaf spraying and/or soil fertilization—were investigated in soybean plants under different edaphoclimatic conditions (field and greenhouse). Beneficial effects of the Soil, Soil + Leaf and Seed + Leaf treatments were observed, with gains of 7 to 20% in biological nitrogen fixation, 1.5-fold in ureides, 14% in shoot dry weight and yield increases of up to 1161 kg ha^−1^. All the Ni application methods resulted in a 1.1-fold increase in the SPAD index, a 1.2-fold increase in photosynthesis, a 1.4-fold increase in nitrogenase, and a 3.9-fold increase in urease activity. Edaphoclimatic conditions exerted a significant influence on the treatments. The integrated approaches, namely, leaf application in conjunction with soil or seed fertilization, were more effective for enhancing yield in soybean cultivation systems. The determination of the ideal method is crucial for ensuring optimal absorption and utilization of this micronutrient and thus a feasible and sustainable management technology. Further research is warranted to establish official guidelines for the application of Ni in agricultural practices.

## Introduction

The proper management of plant nutrients in production systems is one of the main approaches used to achieve agricultural sustainability^1,2^. In recent decades, the use of microorganisms as a biological alternative to mineral fertilization has become increasingly popular in agriculture^3–5^. The significance of nitrogen (N) to crop survival, growth and productivity has been documented^6^. Diazotrophic bacteria promote biological nitrogen fixation (BNF), providing an efficient means of supplying N to plants. In some plant species, BNF can even completely replace nitrogen fertilizers^7,8^. In agricultural systems, approximately 80% of the nitrogen gas (N_2_) fixed comes from symbiosis with plants of the *Fabaceae* family^9–11^. According to the Food and Agriculture Organization of the United Nations^12^, the leguminous soybean plant (*Glycine max* [L.] Merrill) is the most widely cultivated plant species of this botanical family in the world and is responsible for the most efficient BNF process ever reported^13^. This phenomenon results in a high protein content of approximately 40% in the grains; consequently, this plant constitutes a significant nutritional component in the diets of both humans and animals, in addition to its extensive industrial applications^14^.

With the emergence of increasingly productive and nutrient-demanding genotypes of this legume, there is concern about the ability of diazotrophic bacteria to meet the N demand of soybean plants^15,16^. In such a case, micronutrients play a crucial role in plant growth under both normal and stressed conditions. Nickel (Ni), a micronutrient, may be an ideal ally for enhancing the symbiosis between N_2_-fixing bacteria and soybeans. Recent publications have demonstrated the benefits of fertilization with this micronutrient on N metabolism, particularly due its ability to increase the efficiency of the BNF process^17–19^.

Nickel is the latest mineral element to be included on the list of micronutrients necessary for plants^20–22^ because it activates the metalloenzyme urease^23^, which is responsible for the hydrolysis of urea into two molecules: ammonia and carbon dioxide (CO_2_). Thus, Ni plays a direct role in N metabolism, consequently benefiting a wide range of physiological processes and plant development^24,25^. Recent studies on the association between the deficiency of this micronutrient and antioxidant metabolism, ureides, amino acids, and organic acids suggest that Ni may play additional roles in plant nutrition^26–28^. In eubacteria, archaea, and fungi, Ni is an essential catalytic cofactor of at least eight enzymes in addition to urease, with NiFe-hydrogenase being particularly noteworthy^29^. This enzyme promotes the oxidation of hydrogen gas (H_2_) into protons and electrons in N_2_-fixing bacteria^30^, which may promote energy cycling and increase the efficiency of the BNF process^31^.

Under tropical conditions, Freitas et al*.* reported a hidden (latent) deficiency of Ni in a wide range of modern soybean genotypes grown in field^32^. According to the authors, the deficiency of this micronutrient, due to its low availability in some soils, prevents the maximum productive potential of the genotypes from being expressed. This issue had already been raised by Wood, who suggested that Ni deficiency in many plant species does not present visible symptoms^33^. These results highlight the need for Ni fertilization in agricultural crops. However, the need for its addition to cultivated species requires further investigation.

In this context, the key point for overcoming hidden Ni deficiency in sustainable soybean cultivation areas is the identification of the ideal application method. The use of the most appropriate method, i.e., seed treatment, leaf spraying or soil fertilization, is essential to ensure the absorption and ideal use of this micronutrient by plants, consequently leading to greater nutrient use efficiency, yield and profitability of agricultural systems^34–36^. According to these authors, each application method has advantages and limitations. For example, the supply of micronutrients via the soil may require the application of high doses of fertilizers due to the low efficiency of recovery of some nutrients by plants; this occurs because nutrients can be lost in the soil by processes such as leaching, erosion, and immobilization, among others. A benefit of this approach is that such doses may provide a residual effect for subsequent crops. Nevertheless, in the medium and long term, metals can accumulate in the trophic system^37^. Seed treatment with micronutrients is an option that would reduce the dose, which may reduce fertilizer costs and promote plant nutrition in the early growth phase when the poorly developed root system limits the absorption of nutrients from the soil. However, seed treatment can cause phytotoxicity to seedlings; this is especially true for micronutrients, for which there is a small difference between the appropriate and toxic doses^38^. Finally, leaf spraying is an application method already used by farmers for the management of micronutrients. Usually, the sprayed doses are lower than those used in the soil application method to prevent toxicity. Nevertheless, this method can increase production costs due to the need for several applications during the crop cycle to reach an adequate dose and because it does not provide nutrients at the beginning of plant development. These application methods can be used in combination, which could help resolve the aforementioned limitations.

The relative abilities of different Ni application methods to promote the growth and yield of soybean plants are still poorly understood. To our knowledge, this is the first study to investigate the effects of individual and combined Ni application methods—seed treatment, leaf spraying, and/or soil fertilization—on the production of soybean plants grown under different edaphoclimatic conditions and in a greenhouse, with an emphasis on the efficiency of the BNF process and N metabolism.

## Results

### Experiments under field conditions

**Figure 1:**
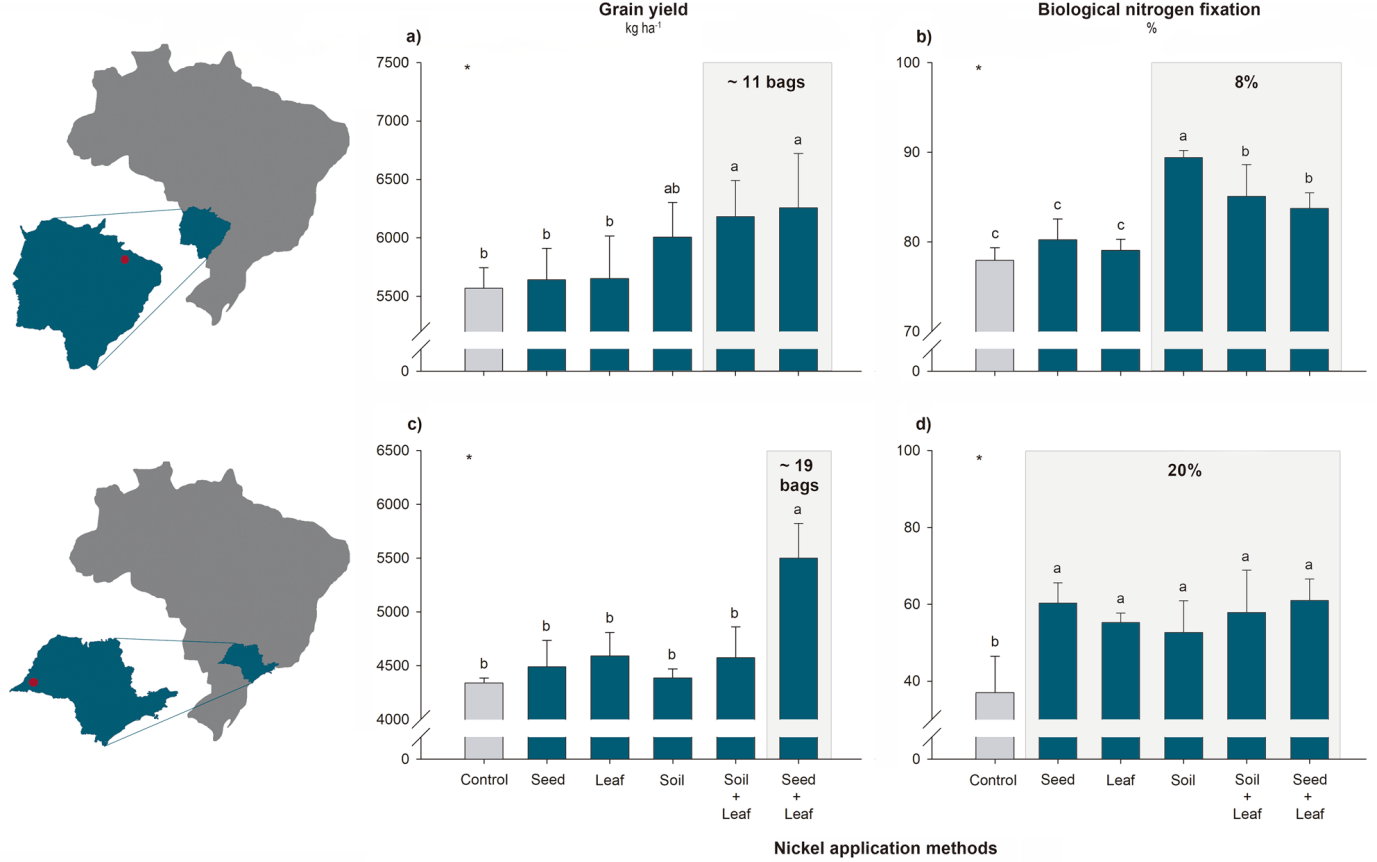
Effects of different Ni application methods on soybean plants grown under field conditions in edaphoclimatic environments 1 (**a**,**b**) and 2 (**c**,**d**). Measurements were taken at the full maturity stage (R8) to assess yield variables (**a**,**c**) as well as at the full flowering stage (R2) to assess biological N_2_ fixation efficiency (**b**,**d**). *Significant according to the *F* test at *p* < 0.05. The mean values and standard deviations were calculated from four replicates. Different letters indicate significant differences according to Fisher’s least square difference (LSD) test at *p* < 0.05. One bag of soybeans is equivalent to 60 kg. The gray rectangles highlight the most responsive treatments.

In edaphoclimatic environment 1, with sandy clay soil (Table 1), the combined treatments, i.e., Soil + Leaf and Seed + Leaf, were the only ones that led to significant increases in the yield indices compared to that in the control treatment, with an average increase in grain yield of 653 kg ha^−1^ (corresponding to ~ 11 bags, with 60 kg per bag) (Fig. 1a). The BNF efficiency followed the yield trend; that is, the highest yield occurred in the treatments with the highest BNF values, with an average increase of 8% compared to that of the control (Fig. 1b). For edaphoclimatic environment 2, with sandy loam soil (Table 1), only the Seed + Leaf treatment responded to Ni fertilization, with an average increase in grain yield of 1161 kg ha^−1^ (~ 19 bags) (Fig. 1c). In this environment, all the Ni-treated plants had significantly greater BNF than did the plants in the control treatment, with a 20% increase in the N_2_ fixation process (Fig. 1d).

**Table 1:** Characterization of two environments with distinct soil and climate conditions—Chapadão do Sul in Mato Grosso do Sul (edaphoclimatic environment 1) and Presidente Bernardes in São Paulo (edaphoclimatic environment 2). In addition to the two experiments conducted under field conditions, a third experiment was conducted in a greenhouse in pots with soil.

Property	Method/extractant^39^	Units	Field		Greenhouse
			Edaphoclimatic environment 1	Edaphoclimatic environment 2	
**Soil chemistry**					
Organic matter	Dichromate/colorimetry	g kg^−1^	29.1	16.5	14.3
pH	Calcium chloride	–	4.7	5.8	4.7
Al	Potassium chloride	cmol_c_ kg^−1^	< 0.1	< 0.1	0.5
Al + H	Calcium acetate, pH 7	cmol_c_ kg^−1^	4.0	1.9	2.6
P	Mehlich-1	mg kg^−1^	15	20.3	1.1
K	Mehlich-1	mg kg^−1^	107.9	58.6	9.2
Ca	Potassium chloride	cmol_c_ kg^−1^	2.6	1.5	< 0.1
Mg	Potassium chloride	cmol_c_ kg^−1^	0.5	0.7	< 0.1
S	Calcium phosphate	mg kg^−1^	4.9	3.4	6.3
B	Hot water	mg kg^−1^	0.2	0.3	< 0.1
Cu	Mehlich-1	mg kg^−1^	1.7	1.1	1.2
Fe	Mehlich-1	mg kg^−1^	35.5	14.6	130.8
Mn	Mehlich-1	mg kg^−1^	13.7	3.9	2.4
Zn	Mehlich-1	mg kg^−1^	5.5	0.6	< 0.1
Available Ni	Mehlich-1	mg kg^−1^	< 0.2	< 0.2	< 0.2
Pseudototal Ni	EPA 3050 B	mg kg^−1^	0.79	0.58	4.65
**Soil physics**					
Clay	Hydrometer	g kg^−1^	480	170	150
Silt	Hydrometer	g kg^−1^	60	50	20
Sand	Hydrometer	g kg^−1^	460	780	830
Soil texture^40^	–	–	Sandy clay	Sandy loam	Sandy loam
**Soil classification**					
Brazilian—SiBCS^40^	–	–	*LVAd*	*PVd*	*LVAd*
FAO^41^	–	–	Ferralsols	Acrisols, Lixisols, Alisols	Ferralsols
Soil taxonomy^42^	–	–	Oxisols	Ultisols, Oxisols	Oxisols
**Climatic characteristics**					
Climate classification^43^	–	–	Am	Aw	–
Elevation^43^	–	m	905	310	–
Annual rainfall^43^	–	mm	1600–1900	1000–1300	–
Annual average temperature^43^	–	°C	20–24	20–24	–

*SiBCS* Brazilian Soil Classification System, *FAO* Food and Agriculture Organization, *LVAd* Typical Dystrophic Yellow‒Red Latosol, *PVd* Typical Dystrophic Red Argisol, *Am* tropical zone with a monsoon climate, *Aw* tropical zone with a dry winter. Chemical properties of soils before correction of acidity and fertilization.

The yield values followed the same pattern as the leaf Ni concentrations (Table 2). In edaphoclimatic environment 1, the average concentrations ranged from 0.19 to 0.41 mg of Ni kg^−1^; the highest concentrations were observed in the Soil + Leaf and Seed + Leaf treatments, with 2.2- and 1.8-fold increases relative to the control, respectively. The Ni concentrations in the grains did not differ between treatments, with an average value of 0.35 mg kg^−1^. In edaphoclimatic environment 2, the average concentrations in the leaves ranged from 0.43 to 1.31 mg of Ni kg^−1^, and the concentration in the Seed + Leaf treatment was threefold greater than that in the control. The Ni concentrations in the grains in edaphoclimatic environment 2 ranged from 0.94 to 1.72 mg kg^−1^. The Seed + Leaf treatment and the control had the lowest Ni values, indicating a probable effect of dilution due to a greater grain yield (Fig. 1c and Table 2). In both edaphoclimatic environments, the N concentrations did not differ between treatments, with average values of 52.43 g kg^−1^ in leaves and 63.21 g kg^−1^ in grains (considering the values of both environments) (Table 2).

**Table 2:** Effects of different Ni application methods on soybean plants grown in the field and under greenhouse conditions. Measurements were taken at the full flowering stage (R2) to assess N and Ni concentrations in leaves and nodules as well as at the end of the cycle (R8) to assess Ni concentrations in grains. The available and pseudototal concentrations of Ni in the soil after cultivation were also measured.

Ni application method	Plant						Soil (mg kg^−1^)	
	N concentration (g kg^−1^)			Ni concentration (mg kg^−1^)			Available Ni^a^	Pseudototal Ni^b^
	Leaf	Nodule	Grain	Leaf	Nodule	Grain		
**Field—edaphoclimatic environment 1**								
Control	49.53 ± 1.79	–	60.98 ± 1.38	0.19 ± 0.01 d	–	0.32 ± 0.04	< 0.2	0.79 ± 0.16 e
Seed	53.24 ± 3.37	–	63.75 ± 1.05	0.25 ± 0.05 cd	–	0.30 ± 0.02	< 0.2	1.28 ± 0.16 cd
Leaf	53.50 ± 0.99	–	61.01 ± 1.89	0.21 ± 0.02 d	–	0.36 ± 0.02	< 0.2	1.20 ± 0.10 d
Soil	52.71 ± 3.96	–	62.35 ± 2.61	0.28 ± 0.05 c	–	0.34 ± 0.01	< 0.2	1.65 ± 0.13 b
Soil + Leaf	49.61 ± 3.64	–	59.72 ± 2.76	0.41 ± 0.05 a	–	0.40 ± 0.01	< 0.2	2.05 ± 0.11 a
Seed + Leaf	54.83 ± 0.65	–	62.72 ± 1.14	0.34 ± 0.02 b	–	0.36 ± 0.03	< 0.2	1.44 ± 0.08 c
*F* test	ns	–	ns	*	–	ns	ns	*
**Field—edaphoclimatic environment 2**								
Control	51.83 ± 2.35	–	65.23 ± 1.00	0.43 ± 0.03 d	–	1.08 ± 0.15 de	< 0.2	0.58 ± 0.12 b
Seed	52.53 ± 2.81	–	64.88 ± 1.60	0.43 ± 0.03 d	–	1.20 ± 0.17 cd	< 0.2	0.62 ± 0.15 b
Leaf	56.81 ± 1.76	–	63.53 ± 1.45	1.03 ± 0.05 b	–	1.28 ± 0.07 c	< 0.2	1.07 ± 0.08 a
Soil	50.67 ± 1.84	–	65.13 ± 1.00	0.70 ± 0.17 c	–	1.49 ± 0.10 b	< 0.2	0.97 ± 0.39 a
Soil + Leaf	51.92 ± 4.75	–	64.95 ± 2.22	1.10 ± 0.10 b	–	1.72 ± 0.16 a	< 0.2	1.10 ± 0.04 a
Seed + Leaf	51.93 ± 3.80	–	64.32 ± 1.17	1.31 ± 0.17 a	–	0.94 ± 0.15 e	< 0.2	1.15 ± 0.10 a
*F* test	ns	–	ns	*	–	*	ns	*
**Greenhouse**								
Control	27.93 ± 4.26	56.27 ± 1.47	–	0.36 ± 0.06 c	2.69 ± 1.27 d	–	< 0.2 c	4.65 ± 0.86 b
Seed	28.05 ± 2.73	55.30 ± 2.41	–	0.46 ± 0.08 c	4.27 ± 0.76 c	–	< 0.2 c	4.96 ± 2.00 b
Leaf	27.30 ± 0.22	55.17 ± 1.20	–	0.67 ± 0.10 b	2.19 ± 0.25 cd	–	< 0.2 c	4.41 ± 0.96 b
Soil	29.15 ± 2.05	55.35 ± 0.95	–	0.60 ± 0.06 b	8.16 ± 1.23 b	–	0.24 ± 0.01 b	7.47 ± 0.36 a
Soil + Leaf	28.68 ± 1.84	56.68 ± 2.21	–	0.83 ± 0.18 a	11.27 ± 1.60 a	–	0.27 ± 0.02 a	6.83 ± 0.53 a
Seed + Leaf	27.65 ± 1.84	56.80 ± 0.71	–	0.69 ± 0.03 ab	4.34 ± 0.33 c	–	< 0.2 c	5.07 ± 0.93 b
*F* test	ns	ns	–	*	*	–	*	*

^a^Mehlich-1. ^b^EPA 3050 B. ^ns^Not significant. *Significant according to the *F* test at *p* < 0.05. Mean values and standard deviations of four replicates. Different letters indicate significant differences according to Fisher's least square difference (LSD) test at *p* < 0.05.

In the soil, the available Ni concentration was < 0.2 mg kg^−1^ after soybean cultivation in both studied environments (Table 2). The pseudototal Ni concentration in the soil ranged from 0.79 to 2.05 mg kg^−1^ in edaphoclimatic environment 1, with the values in the soil treatments increasing 2.3-fold compared to the initial/control concentrations. In environment 2, the pseudototal values ranged from 0.58 to 1.15 mg kg^−1^, with the Leaf, Soil, Soil + Leaf and Seed + Leaf treatments leading to a 1.8-fold increase compared to the initial/control concentrations (Table 2).

### Greenhouse experiment

**Figure 2:**
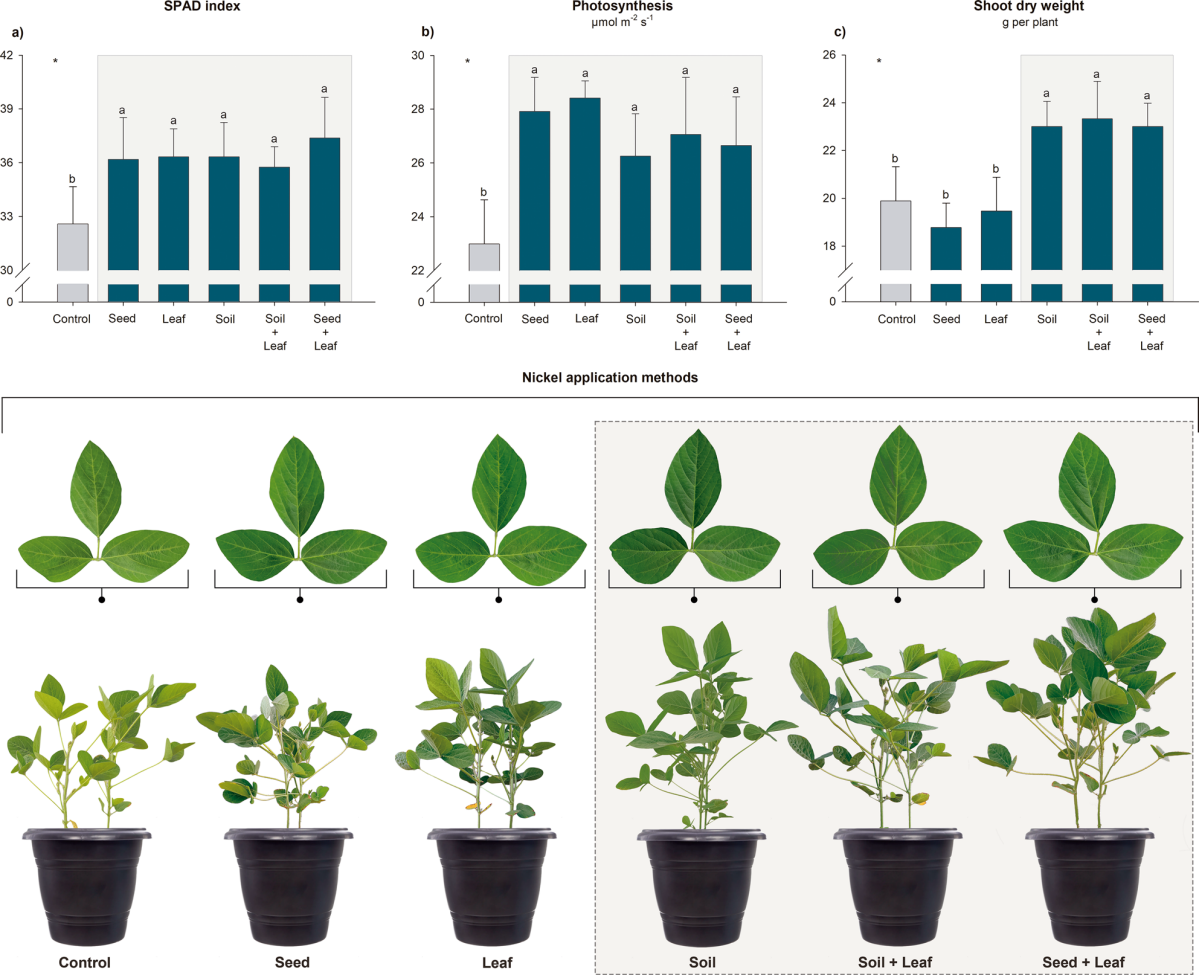
Effects of Ni application methods on soybean plant growth under greenhouse conditions. Measurements were taken at the full flowering stage (R2) for assessment of the SPAD index (**a**) and photosynthesis (**b**) and at the grain filling stage (R6) for assessment of the shoot dry weight (**c**). A visual comparison of the plants and details of their leaves revealed increased growth and a green color in the plants treated with Ni. *Significant according to the *F* test at *p* < 0.05. The mean values and standard deviations were calculated from four replicates. Different letters indicate significant differences according to Fisher’s least square difference (LSD) test at *p* < 0.05. The gray rectangles highlight the most responsive treatments.

**Figure 3:**
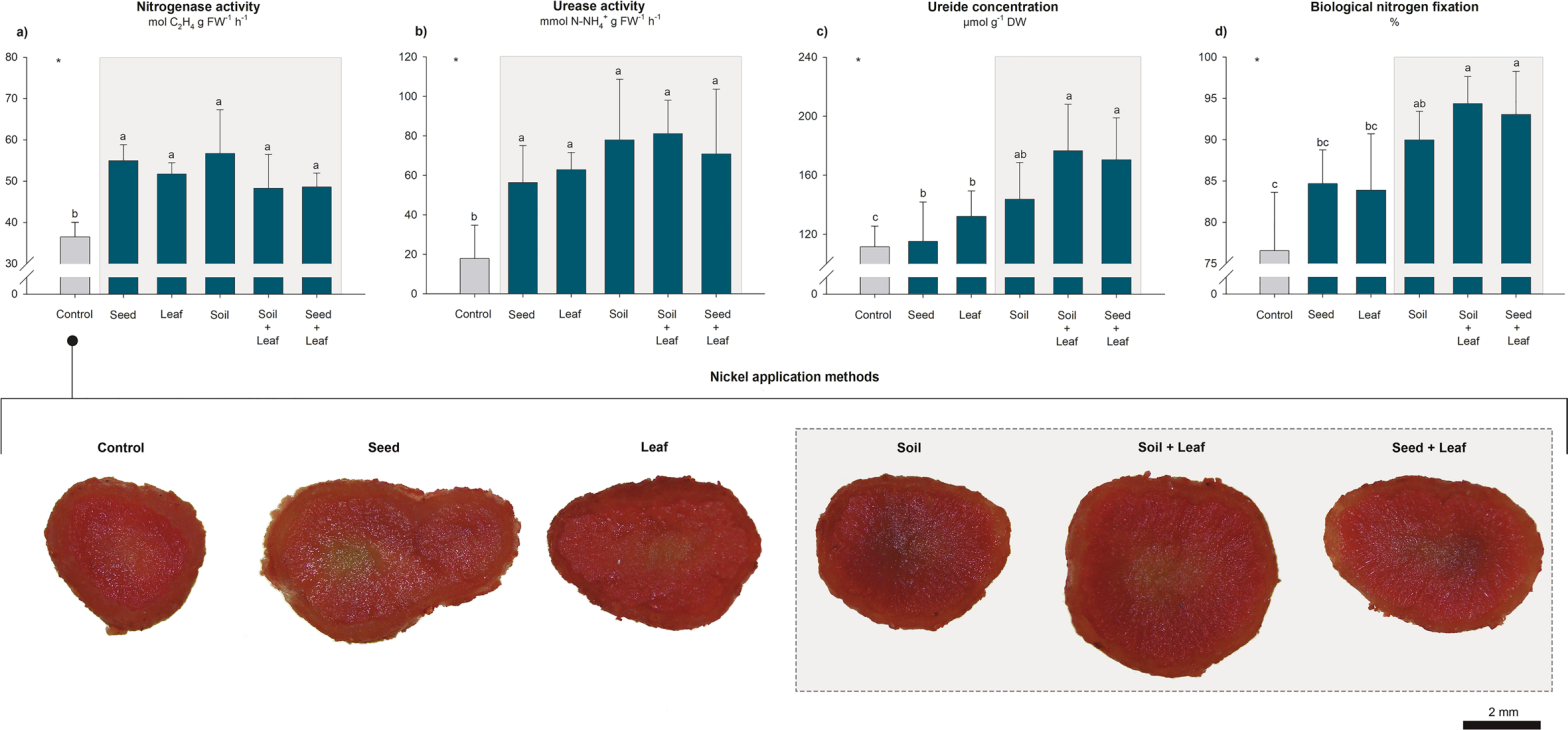
Effects of Ni application methods on the N metabolism of soybean plants grown under greenhouse conditions. Measurements were taken at the full flowering stage (R2) and included nitrogenase activity in root nodules (**a**), leaf urease activity (**b**), leaf ureide concentration (**c**), and efficiency of biological N_2_ fixation (**d**). Cross-sections of root nodules were compared between treatments based on the intensity of red color relative to their activity. *Significant according to the *F* test at *p* < 0.05. The mean values and standard deviations were calculated from four replicates. Different letters indicate significant differences according to Fisher’s least square difference (LSD) test at *p* < 0.05. The gray rectangles highlight the most responsive treatments.

Compared with the control treatment, the Soil, Soil + Leaf and Seed + Leaf treatments promoted greater growth of soybean plants, corresponding to an increase of ~ 14% (Fig. 2c). Similar trends were observed for the leaf concentration of ureides and BNF efficiency, for which the Soil + Leaf and Seed + Leaf treatments stood out, followed by the Soil treatment, with a 1.5-fold average increase in the ureide concentration and a 16% increase in the BNF efficiency compared to those in the control (Fig. 3c,d). These results were consistent with the leaf Ni concentration, which ranged from 0.36 to 0.83 mg kg^−1^, with higher values in the plants in the Soil + Leaf and Seed + Leaf treatments than in the control, corresponding to a 2.3-fold average increase in the Ni concentration (Table 2).

All the treatments that involved the application of Ni promoted significant increases in the growth and N metabolism parameters compared to those in the control, although there were no differences among treatments. There was a 1.1-fold average increase in the SPAD index, a 1.2-fold average increase in photosynthesis, a 1.4-fold average increase in nitrogenase activity and a 3.9-fold average increase in urease activity (Figs. 2a,b, 3a,b). The N concentrations in leaves and in root nodules did not differ significantly between treatments, with average values of 28.1 and 55.9 g kg^−1^, respectively (Table 2).

The Ni concentration in the root nodules was greater in the treatments with soil application methods than in the control, ranging from 2.2 to 11.3 mg kg^−1^, corresponding to increases of up to 5.1-fold (Table 2). This behavior was directly related to the available and pseudototal Ni concentrations in the soil. After plant cultivation, the Soil and Soil + Leaf treatments had average available and pseudototal Ni concentrations of 0.25 and 7.15 mg kg^−1^. The pseudototal concentration of Ni in the soil increased 1.6-fold compared to that in the initial/control settings (Table 2).

**Figure 4:**
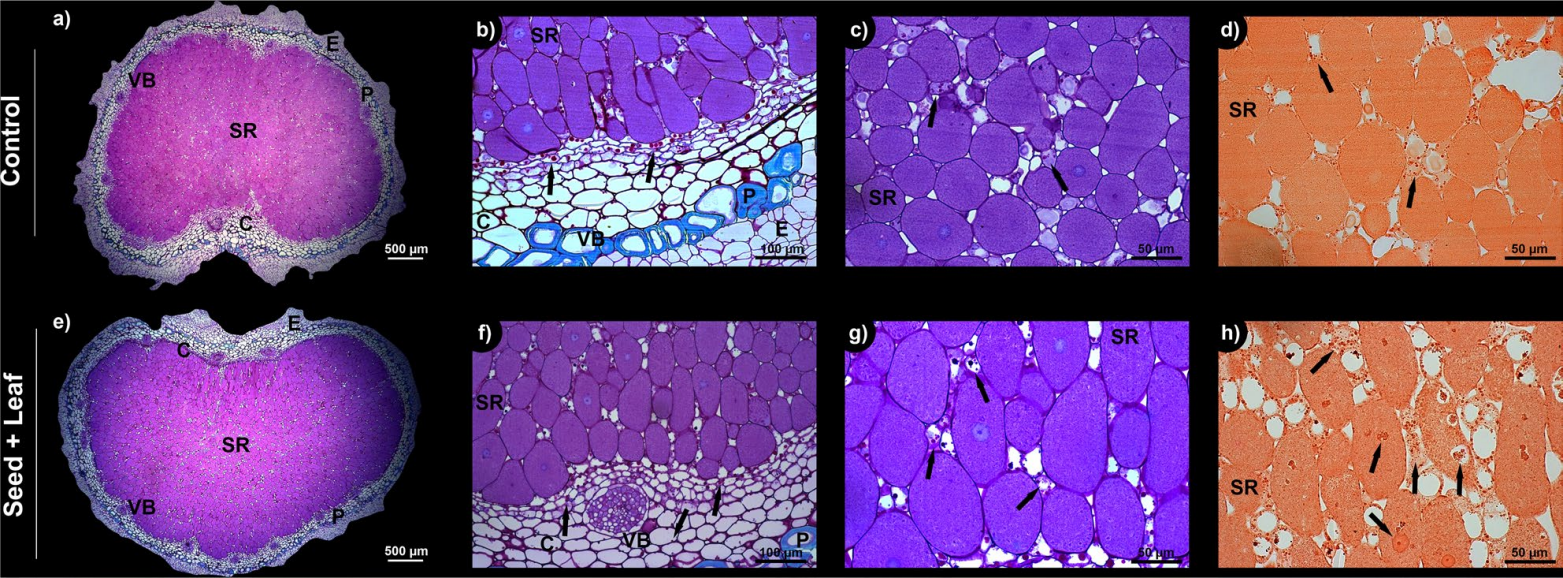
Light microscopy of cross-sections of root nodules from soybean plants in the control (**a**–**d**) and Seed + Leaf (**e**–**h**) treatments at the full flowering stage (R2) in the greenhouse experiment. Positive reactions for periodic acid-Schiff, toluidine blue, and xylidine ponceau reagents were observed, with magenta staining indicating total polysaccharides (including starch grains and mucilage), green staining indicating fibers, and red staining indicating total proteins. Polysaccharides (**f**,**g**) and proteins (**h**) were more abundant in the Seed + Leaf treatment than in the control (**b**–**d**). No anatomical differences were observed in the nodules. The black arrows highlight the accumulation points of the analyzed compounds. *SR* symbiotic region, *C* cortex, *VB* vascular bundles, *P* periderm, *E* epidermis.

The imaging of root nodules by light microscopy revealed positive reactions for the periodic acid-Schiff, toluidine blue and xylidine ponceau reagents, with magenta staining for total polysaccharides, including starch grains and mucilage; green staining for fibers; and red staining for total proteins. The intensity of the reaction and the identification of polysaccharide accumulation sites and protein bodies were greater in the Seed + Leaf treatment than in the control (Fig. 4). These results corroborate the quantitative data previously presented for the upregulation of N metabolism in soybean as a function of Ni application (Fig. 3). Anatomical differences in the nodular tissues were not observed.

## Discussion

A review of studies on Ni fertilization in soybean plants from the last 15 years showed that the methods for applying this micronutrient have never been compared; rather, they have been tested only separately. In general, individual Ni fertilization provides beneficial effects when applied at agronomic doses (Table 3). The benefits commonly reported for Ni application via seed treatment, leaf spraying or soil fertilization include a higher germination rate, seed quality and plant growth; greater photosynthetic activity; more efficient disease control; and positive effects on N metabolism, which are mainly related to BNF (nodulation and flavonoid metabolism). These benefits were or were not associated with increases in yield (Table 3). These results corroborate the data reported in our study, in which the Ni application methods did not cause toxicity, benefiting the nutritional status (Table 2), biological N_2_ fixation (Figs. 1b,d, 3d and 4), N metabolism (Fig. 3) and plant growth parameters (Fig. 2) both in association with and independent of responses in shoot growth and yield of soybean plants (Figs. 1a,c and 2c).

**Table 3:** Compilation of studies from the last 15 years on the approaches used to apply the micronutrient Ni through seed treatment, leaf spraying or soil fertilization in soybean plants.

Method	Ni dose	Substrate/environment	Leaf Ni concentration	Grain yield	Beneficial responses	Reference
			(mg kg^−1^)^a^			
**Seed**	45 mg kg^−1^	Soil Greenhouse	~ 1.22	Increase of 84%	12% increase in biological N_2_ fixation N metabolism Plant growth	^17^
	360 mg kg^−1^	Germination chamber	6.1–12.3 (seed)	Not evaluated	Germination rate Length of the seedling root	^38^
**Leaf**	132 g ha^−1^	Field	0.1–8.3	No response	–	^44^
	20 g ha^−1^	Field	~ 8.7	Increase	Nutritional status Seed quality	^45^
	40 g ha^−1^	Field	~ 14–16	No response	Reduction in powdery mildew severity Stimulation of antioxidant metabolism N metabolism	^46^
	23.2 g ha^−1^	Field	Not evaluated	Increase of 430 kg ha^−1^	N extraction for the grains Protein content	^47^
	0.04 g L^−1^	Nutrient solution Substrate Greenhouse	18.5–40.4^48^	Not evaluated	35% reduction in the severity of soybean rust Photosynthetic capacity Concentration of sugars and ethylene	^48–50^
	60 g ha^−1^	Nutrient solution Greenhouse	Not evaluated	Not evaluated	No response	^51^
	60 g ha^−1^	Soil Greenhouse	Not evaluated	Reduction	–	^52^
**Soil**	0.5 mg kg^−1^	Soil Greenhouse	0.75–4.67	No response	N metabolism Organic acids	^53^
	0.5 mg kg^−1^	Soil Greenhouse Field	0.65–2.26	Greenhouse—increase of up to 2.9 g per plant (12 genotypes), no response in 3 genotypes Field—increase of up to 1502 kg ha^−1^ (4 genotypes), no response in 11 genotypes	N metabolism Photosynthesis	^32^
	3.35 mg kg^−1^	Soil Greenhouse	1.8–2.2	Increase of up to 12.8 g per plant	N metabolism Biological N_2_ fixation Photosynthesis Plant growth	^18^
	0.25 mg kg^−1^	Soil Greenhouse	~ 0.25–1.25	No response	N metabolism 25% increase in shoot dry weight	^54^
	5 mg kg^−1^	15 soils Greenhouse	1.21–15.2	Not evaluated	Increase in biomass of up to 29% in 3 soils No response in 12 soils	^55^
	10 mg kg^−1^	Soil Greenhouse	–	No response	–	^56^
	2–3 kg ha^−1^	Field	1.52	Increase of 391–658 kg ha^−1^	N metabolism Biological N_2_ fixation	^37^
	1.5–3.0 mg kg^−1^	Substrate Greenhouse	–	Increase of 19–51% in pods per plant and 15% in seed weight	N metabolism Nodulation Flavonoid metabolism	^19^
	10–200 mg kg^−1^	Soil Greenhouse	–	Increase in seed yield by 39%	Photosynthesis Mineral homeostasis Phytohormones N metabolism Total fatty acid content	^57^

^a^Results of Ni application using the dose presented in column two of this table.

To date, the study by Lavres et al*.* is one of the few studies on the application of Ni via seed treatment in soybean. In this study, the optimal dose of 45 mg Ni kg^−1^ via seeds improved N metabolism, with a 12% increase in BNF associated with increased plant growth^17^. The findings of our study are consistent with those of the previous study in only one environment, edaphoclimatic environment 2, in which an increase of 20% in BNF was found (Fig. 1d). In the greenhouse, other parameters also responded positively to this treatment (Figs. 2 and 3). In contrast to the findings of Lavres et al*.*, no significant increases in plant growth or grain yield were observed with the application of Ni via seeds^17^. Under all the studied conditions, this treatment resulted in leaf Ni concentrations similar to those found in the control, suggesting that this dose probably did not meet the demand for this micronutrient in soybean plants (Table 2). Thus, our data demonstrated the need for more studies to calibrate the Ni dose applied via seeds and demonstrated that the demand for this micronutrient among soybean genotypes may vary considerably. This trend was also observed by Freitas et al*.*, who reported different groups of responsiveness of soybean genotypes to Ni^32^. Recently, Oliveira et al*.* demonstrated that the supply of Ni via seeds using conventional agricultural sources, even at very high doses of 360 mg of Ni kg^−1^, is safe for the development of soybean seedlings if the Ni remains adhered to the seed coat, especially to the hilum^38^. Rather than migrating directly toward the emerging cotyledons, Ni first moves to the soil, where it can then be absorbed by the roots of the seedlings. These results provide promising information about the adequacy of safe Ni doses for agricultural management via seed treatment.

Similar to the results observed for the seed treatment, Ni application via leaves had positive effects on several growth parameters (Fig. 2) and N metabolism (Figs. 1b,d, 3 and 4) but was not associated with increased plant growth or grain yield (Figs. 1a,c and 2c). Our data corroborate the studies by Alovisi et al. and Einhardt et al., in which leaf spraying with Ni did not promote significant responses in plant development^44,51^. In general, the application of doses of up to 60 g of Ni ha^−1^ was safe and did not cause phytotoxicity among the soybean plants (Table 3); however, the beneficial effects of spraying with this micronutrient were observed when the application was associated with some stressful growing conditions, such as the occurrence of fungal diseases and glyphosate toxicity^45,46,48–50^, as well as with concomitant application of other nutrients, such as molybdenum^47^.

Compared with the seed and leaf application methods, studies on soil Ni fertilization are more advanced, with a range of growing environments, soil classes and soybean genotypes having been evaluated previously (Table 3). It has been reported in the literature that this method improves N metabolism in soybean plants^18,19,32,37,53,54,57^, as found in this study (Figs. 1b,d, 3 and 4). These beneficial responses may or may not promote plant growth and grain yield (Table 3)^18,19,32,37,53–57^. Our results confirmed this behavior (Figs. 1a,c and 2c). In the greenhouse experiment, there was greater growth of the shoots of soybean plants (Fig. 2c), which was in contrast to the results observed in edaphoclimatic environments 1 and 2, where despite the greater efficiency of the BNF process (Fig. 1b,d), there were no yield increases (Fig. 1a,c). This response can be explained by the dynamics of Ni in the soil; properties such as pH^37,53,55^, soil particle size^54^ and organic matter^56,58^, which affect its availability to plants; and, consequently, the response to fertilization. The soil in edaphoclimatic environment 2, which was a sandier soil (Table 1), had a coarse grain size and was slightly reactive; therefore, it may have favored the mobility of Ni in the profile, possibly causing its loss by leaching^59^. In the soil of edaphoclimatic environment 1, a clayey soil (Table 1), the sorption of Ni to clay minerals promoted rapid conversion to unavailable pseudototal contents^37^. Therefore, in both studied soils, there was a reduction in Ni availability to plants during the crop cycle. As observed in this study (Table 2), according to Rodak et al*.* the availability of Ni in the soil tends to be low after crop harvest, with a small residual effect of this micronutrient being found when it is supplied at agronomic doses^37^. This suggests that soil Ni management may require year-to-year adjustments to fertilizers, which in the long term may result in high pseudototal levels that are environmentally toxic^37^. The more serious environmental issue in this study, that is, the high pseudototal Ni concentration in the soil, occurred in the greenhouse, where the concentration of Ni in the soil reached 7.47 mg of Ni kg^−1^ (Table 2). Although this value is high, it is still below the threshold for prevention and investigation imposed by Brazilian legislation (30 and 70 mg of Ni kg^−1^, respectively)^60^. Therefore, the levels in our study are considered safe for agricultural soils (Table 2). Notably, Macedo et al*.* and Rodak et al*.* recommended constant monitoring of Ni levels in agricultural areas fertilized with this micronutrient to ensure safe environmental levels^37,56^. Moreover, in a recent study by Zhou et al*.* the use of nanoparticles of Ni reduced potential phytotoxicity concerns^57^.

The application of Ni at soybean sowing via seed treatment combined with leaf spray or soil fertilization combined with leaf spray had unprecedented results; together with the soil treatment, these approaches provided the best responses by soybean plants (Figs. 1, 2, 3 and 4). Because of the beneficial effects of Ni, leaf spraying with Ni can be used to complement seed treatment or soil fertilization; thus, the application of Ni supplied at different phenological stages results in greater absorption efficiency. As demonstrated by Ciampitti et al*.*, nodulation and the BNF process are intensified in the V3/V4 phenological stage (second/third fully expanded trifoliate leaves), with a maximum fixation rate around the time of pod formation^61^; thus, supplying Ni at the beginning of the BNF process, as in this study, may contribute to greater N use efficiency by plants. Moreover, a recent study by Bosse et al*.* revealed that Ni plays a role in enhancing nodulation and ureide metabolism via flavonoid metabolism^19^.

## Methods

### Experimental design

Two experiments were carried out under field conditions. Subsequently, to better understand the results obtained from the field investigations, a third experiment was conducted in a greenhouse in pots with soil.

The treatments consisted of six Ni application methods: (1) Control—no Ni fertilization; (2) Seed treatment (hereafter called Seed); (3) Leaf spraying (Leaf); (4) Soil fertilization (Soil); (5) Soil fertilization with leaf spraying (Soil + Leaf); and (6) Seed treatment with leaf spraying (Seed + Leaf). Nickel fertilization was performed using nickel sulfate (NiSO_4_·6H_2_O) at doses of 2.5 g ha^−1^ for seed treatment^17^, 20 g ha^−1^ for leaf spraying^45^ and 1 kg ha^−1^ for soil fertilization^32^, as reported in previous studies.

In the field experiments, the treatments were distributed in a randomized complete block design with four replicates, totaling 24 plots. The total area of the plot was 15 m^2^, with 6 rows of 6.25 m, equally spaced at 0.4 m. In the greenhouse, the treatments were distributed in a completely randomized design, with four replicates, totaling 48 pots.

### Management of experiments under field conditions

From December 2020 to April 2021, two experiments were carried out under field conditions with the soybean cultivar BMX DESAFIO RR in Brazilian cities with distinct soils and climates—Chapadão do Sul in Mato Grosso do Sul and Presidente Bernardes in São Paulo—referred to as edaphoclimatic environments 1 and 2, respectively. The characteristics of the experimental areas are described in Table 1.

Before the implementation of the experiments, the soil pH of the cultivated areas was corrected by raising the base saturation to 60% with the application of limestone. At sowing, fertilization was performed with 80 kg of P_2_O_5_ ha^−1^ using superphosphate and 40 kg of K_2_O ha^−1^ potassium chloride. To provide an N supply, the seeds were inoculated with 2 mL of N_2_-fixing bacteria (*Bradyrhizobium japonicum* and *Bradyrhizobium elkanii*) containing 7 × 10^9^ colony-forming units per milliliter. In phenological stage V3/V4 (second/third fully expanded trifoliate leaves)^62^, 60 kg of K_2_O ha^−1^ fertilizer was applied and micronutrients and the beneficial element cobalt (Co) were added to the soil in the form of potassium chloride and the commercial products Naturamin^®^ Co/Mo and Amino AgRoss, respectively. Fertilization was performed as recommended by Embrapa Cerrados^63^. In the soil treatments, the Ni solution was applied concomitantly with the fertilizer, which provided other nutrients, at the start of the experiments and was applied to the surface of the sowing line. For the seed treatments, the Ni solution was sprayed directly on the seed surface simultaneously with inoculation. Finally, the Ni solution used in the leaf treatments was sprayed on the plants at the V3/V4 phenological stage. Phytosanitary control was performed whenever necessary.

Samples were collected for plant and soil analysis in the useful area of each plot, i.e., in the two central rows, eliminating 1.0 m from each end. In phenological stage R2—full flowering—the third fully expanded leaf (from top to bottom) was collected from ten plants in each plot. The leaves were mixed to form a composite sample, which was used to determine the concentrations of Ni and N, as well as to evaluate the efficiency of BNF. At phenological stage R8—the end of the cycle when pods had a moisture content of less than 15%, the grains of all plants in the useful area of each plot were harvested to determine the yield and concentrations of Ni and N. During soybean harvest (R8), four soil subsamples were collected per plot at a depth of 0–20 cm. The subsamples were mixed to form a composite sample that was used to determine the available and pseudototal Ni concentrations.

### Management of the greenhouse experiment

In a greenhouse, the soybean cultivar BMX DESAFIO RR was sown in pots (5 L capacity) filled with soil, and two plants were cultivated per pot. The chemical, physical and pedological characteristics of the soil used are described in Table 1.

The soil pH was corrected by raising the base saturation to 60%. A mixture of calcium carbonate and magnesium carbonate at a ratio of 3:1 was added to the soil, which was then homogenized. The soil was incubated for 30 days with moisture at field capacity. Subsequently, at sowing, fertilization with the other macro- and micronutrients and Co (except N and Fe) was performed with a nutrient solution that was mixed into the soil at the following rates: 100 mg of P kg^−1^, 70 mg of K kg^−1^, 15 mg of S kg^−1^, 1 mg of Cl kg^−1^, 1 mg of Mn kg^−1^, 3 mg of Zn kg^−1^, 1 mg of B kg^−1^, 1 mg of Cu kg^−1^, 0.5 mg of Mo kg^−1^ and 0.1 mg of Co kg^−1^. The fertilization sources were calcium phosphate monobasic, potassium phosphate monobasic, magnesium sulfate, zinc sulfate, manganese chloride, boric acid, copper sulfate, ammonium molybdate and cobalt sulfate. Fertilization was performed as recommended by Embrapa Cerrados^63^. The inoculation process and treatments were similar to those described for the field experiments, with the following modifications: in the soil treatments, the Ni solution was added to and mixed with the soil along with the other nutrients at sowing, and for the leaf spraying treatments, the surface of the pots was sealed with plastic film to prevent contact between the Ni solution and the soil. During the experiment, phytosanitary controls were performed whenever necessary, and irrigation was performed daily with deionized water, maintaining soil moisture at field capacity by weighing the pots.

Soybean samples were collected at two distinct phenological stages (R2—full flowering and R6—full grain filling). In R2, the third and fourth fully expanded leaves (from top to bottom) of the plants were collected to determine the concentrations of N and Ni, urease activity, ureide concentration and BNF efficiency. Medium- to large root nodules (> 3.5 mm^2^) were also collected for determination of nitrogenase activity, N and Ni concentrations and light microscopy analysis. In vivo evaluations of the SPAD index and photosynthesis were performed at the same stage. In R6, plant shoots were collected for determination of dry weight, and soil samples were collected for quantification of the Ni concentration.

### Nutritional status of the plant and the soil

To determine the concentrations of Ni in leaves, grains and root nodules, the samples were dried at 65 °C in an oven with air circulation, ground and passed through a 0.5 mm sieve. A 250 mg subsample of the material was digested in an acid solution (75% nitric acid and 25% hydrogen peroxide) in a closed microwave oven system, and the Ni concentration was subsequently quantified via inductively coupled plasma-optical emission spectrometry (ICP-OES) (iCAP 7000 Plus Series, Thermo Scientific, Massachusetts, USA). For N quantification, 10 mg of the sample was weighed in tin capsules and analyzed via an isotopic-ratio mass spectrometer (IRMS) coupled to an elemental N analyzer (ANCA-GSL Hydra 20–20 model, SERCON Co., Crewe, GBR).

The available and pseudototal Ni concentrations in the soil samples were determined using the Mehlich-1 and EPA 3050 B extraction methods, respectively. The samples were air-dried and sieved through a 2-mm mesh. The extract for determination of the available concentrations was obtained from 10 g of soil added to the Mehlich-1 solution (0.05 M hydrochloric acid and 0.012 M sulfuric acid). The sample was agitated for 10 min at 200 RPM, and the supernatant was collected after decanting for 16 h at room temperature^39,64^. To obtain the pseudototal concentration, 0.5 g of soil was digested in nitric acid, hydrochloric acid and hydrogen peroxide in a block digester system following the procedure described by the United States Environmental Protection Agency^65^. The Ni concentrations of the extracts were analyzed using ICP-OES.

Certified reference materials were used for the quality assurance and quality control protocols.

### Biological N_2_ fixation and N metabolism in plants

To evaluate the N_2_ fixation and N metabolism of the plants, the activities of nitrogenase and urease enzymes, the ureide concentration and the BNF efficiency were analyzed.

The activity of the enzyme nitrogenase was determined by the acetylene reduction method^66^. Fifteen root nodules were transferred immediately after collection to a 9 mL flask and hermetically sealed with a rubber stopper. A 1 mL aliquot of acetylene gas was injected into the flasks containing the nodules to induce ethylene synthesis by nitrogenase. After 20 min of incubation, a 1 mL aliquot of the sample was collected and transferred to a new vacuum flask. The determination of the ethylene concentration was performed in a gas chromatograph equipped with a Porapack-N column.

To validate the activity of the enzyme nitrogenase, the efficiency of the BNF process was determined by isotopic variation in the natural abundance of ^15^N (δ^15^N ‰), according to Shearer and Kohl^67^. The following equation was used to calculate the BNF (%): 100 × (δ^15^N reference − δ^15^N soybean)/(δ^15^N reference − B). The reference δ^15^N was obtained from non-N_2_-fixing plants (*Brachiaria brizantha* in the field experiments and *Oryza sativa* in the greenhouse experiment), both of which were cultivated concomitantly under the same experimental conditions as the soybean plants (δ^15^N soybean). The reference δ^15^N value for *Brachiaria brizantha* was 3.59 ‰ (n = 4) in edaphoclimatic environment 1 and 3.40 ‰ (n = 4) in edaphoclimatic environment 2, while the value for *Oryza sativa* was 3.73 ‰ (n = 4). To determine the isotopic composition of δ^15^N ‰, after the samples were collected, the same procedure described above was adopted for the quantification of N in plant tissue. The value of variable B was based on the data obtained by Guimarães et al*.*^68^.

The activity of the urease enzyme was determined by adapting methods described by Hogan et al*.* and McCullough^69,70^. After collection, to obtain the extract, 0.3 g of fresh leaf tissue from the plants was incubated in 5.0 mL of phosphate buffer with urea (pH 7.4) for 1 h at 30 °C to promote the synthesis of ammonium by urease. For the coloring reaction, which occurs as a function of the synthesized ammonium concentration, an aliquot of 250 µL of the extract was collected and transferred to a new tube, to which 2.5 mL of solution 1 (0.1 M phenol and 170 µM sodium nitroprusside) and 2.5 mL of solution 2 (0.125 M sodium hydroxide, 0.15 M sodium phosphate dibasic and 3% sodium hypochlorite) were added. Then, the samples were incubated for 35 min at 37 °C. Urease activity was quantified by colorimetry in a spectrophotometer at an absorbance of 625 nm.

The concentration of leaf ureides (allantoin and allantoic acid) was determined using 0.3 g of dry plant tissue in 10 mL of extractor solution (60% methanol, 25% chloroform and 15% deionized water). For separation of the water-soluble phase, a 6 mL aliquot of the sample was transferred to a new tube, to which 1.5 mL of chloroform and 2.25 mL of distilled water were added. After decantation, an aliquot of 40 µL of the supernatant was analyzed using the method described by Vogels and Van der Drift^71^. In the first stage, 20 µL of 0.33% phenylhydrazine solution and 250 µL of 0.5 M sodium hydroxide solution were added to the tube, and the mixture was subsequently incubated for 8 min at 100 °C. After the samples cooled to room temperature, 250 µL of 0.65 N hydrochloric acid was added, and a new cycle of incubation was performed for 4 min at 100 °C. In the second stage, the samples were kept at room temperature for 5 min, followed by the addition of 250 µL of 0.4 M phosphate buffer (pH 7.0) and 250 µL of phenylhydrazine solution. The samples were incubated on ice in the dark for 5 min. Then, 1.25 mL of previously chilled hydrochloric acid and 250 µL of a 1.65% solution of potassium ferricyanide were added. After the samples were kept at room temperature for 15 min, the ureide concentration was quantified by colorimetry in a spectrophotometer at an absorbance of 535 nm.

### Plant growth

The plant growth parameters were based on grain yield and shoot dry weight, as well as on in vivo measurements of the SPAD index and photosynthesis.

The SPAD index was obtained by quantifying the intensity of the green coloration of the leaves using a portable chlorophyll meter. The CO_2_ assimilation rate, hereafter called photosynthesis, was calculated by measuring the variation in CO_2_ concentration in a closed chamber using a portable infrared gas analyzer (IRGA), LICOR^®^ 6.400XT (LI-COR, Inc., Lincoln, NE, USA). The measurements were performed in the morning, and the photon flux in the chamber was maintained at 1200 µmol m^−2^ s^−1^. The air flow in the sample line was adjusted to 350 µmol s^−1^. During the measurements, the air temperature oscillated between 25 and 26 °C, and the leaf temperature oscillated between 26 and 27 °C.

To determine the yield, the grains were weighed, and their moisture was adjusted to 13%. Grain moisture was determined by oven-drying at 105 ± 3 °C for 24 h^72^. Shoot dry weight was determined by oven-drying with air circulation at 65 °C until water loss stabilized.

### Light microscopy

To better understand and visualize the results obtained in the greenhouse, one of the treatments most responsive to the micronutrient Ni, i.e., the Seed + Leaf treatment, was compared to the control via light microscopy of the root nodules.

After collection, the samples were fixed immediately in Karnovsky’s modified solution (2% glutaraldehyde, 2% paraformaldehyde, 0.001 M calcium chloride in 0.05 M cacodylate buffer, pH 7.2)^73^ under vacuum and refrigerated for 72 h. Then, a series of dehydrations was performed in ethanol, from 30 to 70% v/v, at 1 h for each step, followed by 100% propanol and 100% butanol, with subsequent butanol infiltration (3:1, 1:1 and 1:3 ratios), ending with incorporation into historesin. Polymerization was performed in an infiltration medium and with a hardener at room temperature for 48 h, as recommended by the manufacturer. Cross-sectional histological sections of 4 μm were obtained on a microtome and stained with periodic acid-Schiff reagent and toluidine blue^74^ for analysis of total polysaccharides, starch grains, fibers, and mucilage, while xylidine ponceau was used for total protein analysis^75^. The slides were covered with a coverslip and Entellan^®^. The sections were analyzed, and images were obtained under an upright microscope.

### Statistical analysis

The normality of the data was assessed, followed by analysis of variance. When the results of the analysis of variance were significant, the means were compared using Fisher’s least square difference (LSD) test (*p* < 0.05).

### Research involving plants statement

The authors adhere to the IUCN Policy Statement on Research Involving Species at Risk of Extinction, ensuring strict compliance with all international guidelines and legislation. This research did not use any legally protected species or cause any negative impacts on threatened species. The authors bear a profound moral responsibility for the preservation and enhancement of the survival of these species. This statement is in alignment with the Convention on the Trade in Endangered Species of Wild Fauna and Flora, further emphasizing our commitment to environmental safety and conservation.

## Data Availability

All the data included in this study are available upon reasonable request by contacting the corresponding author.
